# Progress in Scaling up and Streamlining a Nanoconfined, Enzyme‐Catalyzed Electrochemical Nicotinamide Recycling System for Biocatalytic Synthesis

**DOI:** 10.1002/celc.202001166

**Published:** 2020-11-20

**Authors:** Beichen Cheng, Lei Wan, Fraser A. Armstrong

**Affiliations:** ^1^ Department of Chemistry University of Oxford Inorganic Chemistry Laboratory South Parks Road Oxford OX1 3QR

## Abstract

An electrochemically driven nicotinamide recycling system, referred to as the ‘electrochemical leaf’ has unique attributes that may suit it to the small‐scale industrial synthesis of high‐value chemicals. A complete enzyme cascade can be immobilized within the channels of a nanoporous electrode, allowing complex reactions to be energized, controlled and monitored continuously in real time. The electrode is easily prepared by depositing commercially available indium tin oxide (ITO) nanoparticles on a Ti support, resulting in a network of nanopores into which enzymes enter and bind. One of the enzymes is the photosynthetic flavoenzyme, ferredoxin NADP^+^ reductase (FNR), which catalyzes the quasi‐reversible electrochemical recycling of NADP(H) and serves as the transducer. The second enzyme is any NADP(H)‐dependent dehydrogenase of choice, and further enzymes can be added to build elaborate cascades that are driven in either oxidation or reduction directions through the rapid recycling of NADP(H) within the pores. In this Article, we describe the measurement of key enzyme/cofactor parameters and an essentially linear scale‐up from an analytical scale 4 mL reactor with a 14 cm^2^ electrode to a 500 mL reactor with a 500 cm^2^ electrode. We discuss the advantages (energization, continuous monitoring that can be linked to a computer, natural enzyme immobilization, low costs of electrodes and low cofactor requirements) and challenges to be addressed (optimizing minimal use of enzyme applied to the electrode).

## Graphical abstract

**Take a leaf out of their book**: The electrochemical leaf exploits the discovery of rapid and highly efficient electrochemical regeneration of nicotinamide cofactors operating in enzyme cascades entrapped within electrode nanopores. The electrode is produced from commercially available conducting metal oxide nanoparticles. Biocatalytic reactions are simultaneously energized, controlled and monitored. Investigations show that the scale‐up of syntheses to reactor level should be simple, cost‐effective, and ‘green’.

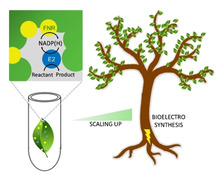

## 1 Introduction

Catalysis by enzymes is widely recognized as a clean, efficient, and enantioselective approach for producing high‐value products in chemical and pharmaceutical industries.[^1^, ^2^, ^3^, ^4^, ^5^, ^6^, ^7^] Many reactions catalyzed by oxidoreductases require nicotinamide cofactors, NAD(P)(H), but were these to be used in stoichiometric amounts, the cost would be prohibitive.[^8^, ^9^] The attraction of enzyme‐catalyzed synthesis has thus driven the development of efficient *in situ* cofactor recycling systems.[^10^, ^11^] Current methods for cofactor regeneration in use industrially include, in particular, the use of glucose dehydrogenase (GDH) or formate dehydrogenase (FDH) in scaled up one‐pot reactions.[^12^, ^13^, ^14^] There are obvious ways to improve biocatalysis for synthesis: aside from immobilizing the enzymes,[^15^, ^16^, ^17^] it would be advantageous to have ways of constantly monitoring the reaction and it would be helpful to minimize use of additional chemicals and enzymes needed for cofactor regeneration. A rapid electrochemical system that comprises nanoconfined components deals with these challenges and also allows the assembly of enzyme cascades that can perform multiple steps in a single reactor.[^18^, ^19^, ^20^, ^21^, ^22^]

**Figure 1:**
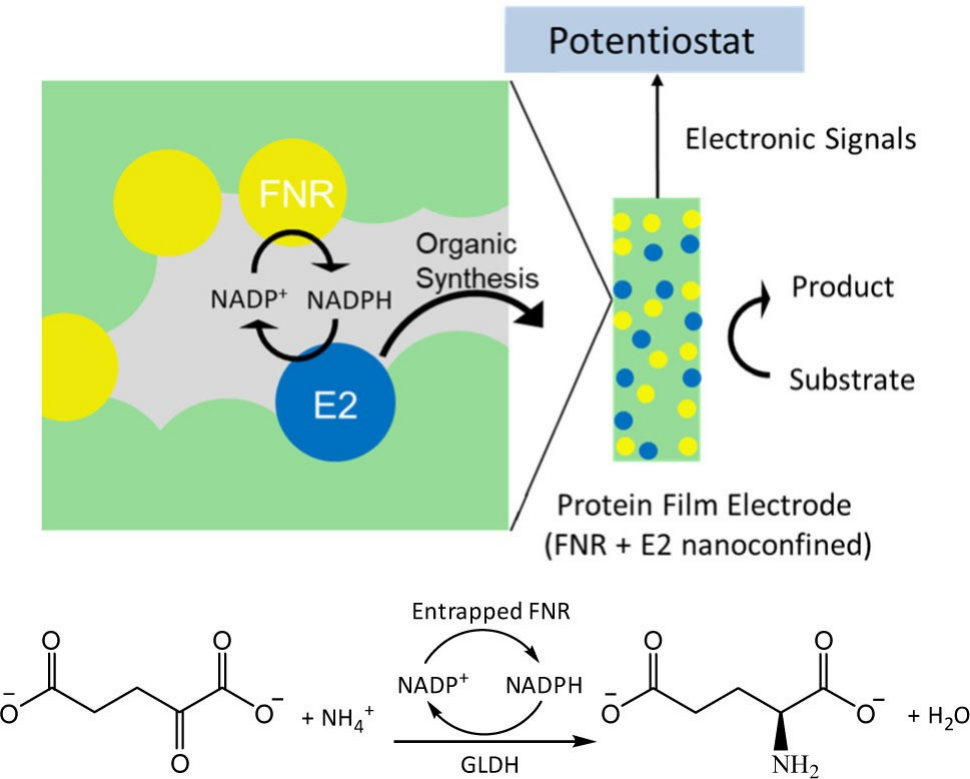
The nanoconfined electrochemical reactor system (top). Conversion of 2‐oxoglutarate to L‐glutamate by L‐glutamate dehydrogenase continuously supplied with NADPH from co‐confined FNR (bottom).

In the ‘electrochemical leaf’, a nanoporous metal oxide electrode is used to entrap at least two enzymes operating in a cascade: one of these is ferredoxin‐NADP^+^ reductase (FNR), the small photosynthetic flavoenzyme responsible for channeling light‐activated electrons into biosynthesis;[^23^, ^24^] the second (E2) can be any of hundreds of NADP(H)‐dependent dehydrogenases. The concept is depicted in Figure 1A. The resulting material is abbreviated (FNR+E2)@MO/support, where MO is a conducting metal oxide such as indium tin oxide (ITO) or fluorine tin oxide (FTO) and the support is typically carbon or titanium. Electrophoretic deposition of commercially available metal oxide nanoparticles on the support generates nanospace in a very simple and natural way, as the channels and pockets formed by irregular packing yield sites for enzyme binding and confinement while maintaining access for solvent and small molecules. Imaging by SEM and TEM reveals that the layer, varying between 1 and 3 μm in depth depending on deposition time, is comprised of nanoparticles <50 nm across that have aggregated to generate dense, random pores with widths 5–100 nm.^[21]^

The nanopores lead to a high local concentration of the enzymes and restrict the escape of NADP(H) or (in an extended cascade) intermediates that are needed for the next stage.[^21^, ^22^] The resulting overall rate and progress of a biocatalytic run are observed directly through the current that flows and the charge that is passed, respectively. The (FNR+E2+…)@ITO/support material thus forms the basis for an inexpensive, easily‐accessible “plug‐in” device able to drive, interactively, a potentially unlimited number of organic reactions depending on the identity of E2 and further enzymes.

To date, the electrochemical leaf has been studied with typical solution volumes in the 2–4 mL range, to explore the effects of coupling with different E2 enzymes at the analytical level and measure the kinetics of the reactions involved.[^19^, ^20^, ^21^, ^25^] It was therefore important and timely to establish the feasibility of scaling up the existing electrochemical leaf system to achieve biocatalysis that could be useful for synthesis of high‐value chemicals that otherwise require separate multiple steps; importantly, identifying both advantages and disadvantages of a biocatalysis system that differs in several ways from those currently used. This article describes the results we have obtained using a simple model reaction, the intention being to focus on the performance of the system rather than of the enzymes and gauge the tolerance to practical variability in conditions. We used L‐glutamate dehydrogenase (GLDH) as the exemplar coupling enzyme: it catalyzes a reductive amination reaction (Figure 1B) in which 2‐oxoglutarate is converted to L‐glutamate in the presence of NH_4_^+^ and NADPH. The enzyme and its reaction are well studied and both reactant and product are easily acquired and quantified.

## 2 Results

### 2.1 The Enzymes

Ferredoxin NADP^+^ reductase was prepared as described below in the Experimental Section.^[18]^ Recombinant *E.coli* L‐glutamate dehydrogenase was prepared as described in Supporting Information. Apart from one experiment, enzyme catalysis was studied at pH 8.0 using [tris(hydroxymethyl)‐methylamino]propanesulfonic acid (TAPS) as buffer.

### 2.2 The Electrodes

The preparation of enzyme‐modified electrodes consists of two stages. First, the support material is coated with ITO nanoparticles creating a highly porous conductive layer. Next, the enzymes are loaded within the pores of the ITO layer.

#### 2.2.1 Electrode Fabrication

Two ITO coating methods have been used in previous work, namely electrophoretic deposition and manual pasting/calcination.[^18^, ^26^] Electrophoretic deposition (EPD) of commercially available ITO nanoparticles (Sigma‐Aldrich <50 nm particle size) is most convenient when dealing with regular shapes such as Ti foil or foam, whereas pasting followed by calcination is more suitable for irregular‐shaped electrode supports such as Ti tubes, although less control is obtained over the amount of ITO loaded. The procedures are described in Supporting Information, where Figures S1–S3 show that the changes in appearance and capacitance following ITO deposition on Ti are comparable for both methods. In this study, in which we focused entirely on Ti foil, we used the EPD method to coat each side. The overall electrode surface was increased by using multiples of centrally wired individual electrodes.

#### 2.2.2 Enzyme Loading

Two methods of enzyme loading have been used in past studies. In the drop‐cast method, a small volume of a concentrated enzyme solution (typically 8 μL cm^−2^ of electrode surface area) is dropped directly onto the surface of each ITO/support electrode (both sides when using Ti foil). A short period of time (usually 20–30 minutes) is allowed for the enzymes to bind, after which the electrode is rinsed thoroughly in ultrapure water to remove any unbound material. For the dilute solution method, the ITO/support electrode is placed in a dilute enzyme solution which is stirred for an extended period of time (typically, 4–18 hours) to allow for adsorption, before removing the electrode and rinsing. The process of FNR and E2 loading can be simultaneous as a mixture, or stepwise using the same or separate methods.

### 2.3 Effect of Varying the Ratio of the Two Enzymes

**Figure 2:**
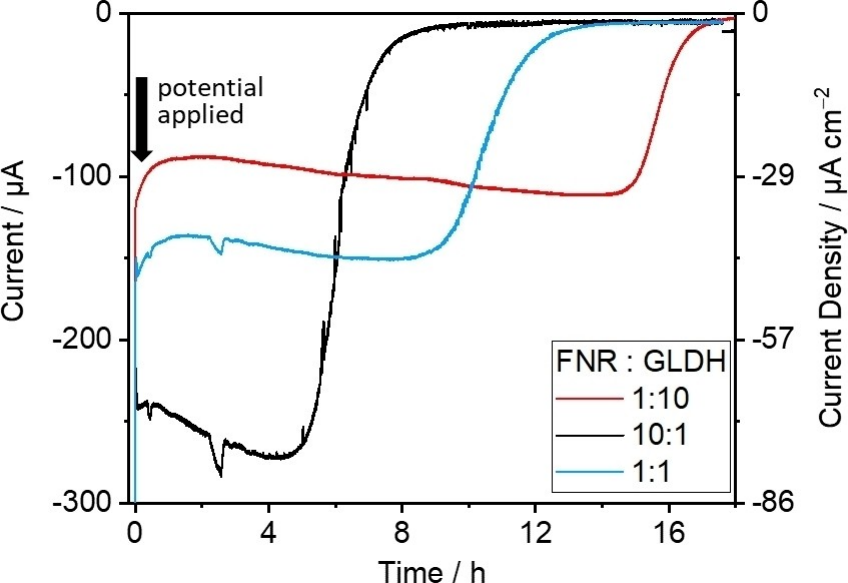
GLDH‐catalyzed reductive amination comparing different FNR:GLDH ratios applied to the ITO/Ti foil electrode. Conditions: [2‐oxoglutarate]=8 mM, [NH_4_Cl]=16 mM, [NADP^+^]=20 μM, buffer: 0.10 M TAPS pH 8.0; temperature: 25 °C, *E* (vs. SHE): −0.48 V; mixing by stirrer bar at 400 rpm; reactor volume: 4 mL; electrode: 2.5 cm×0.7 cm Ti foil, both sides=3.5 cm^2^. Enzyme amounts applied by drop‐casting: 17.02 nmol FNR+1.70 nmol GLDH (10 : 1); 9.35 nmol FNR+9.35 nmol GLDH (1 : 1); 1.70 nmol FNR+17.02 nmol GLDH (1 : 10).

A factor that is important to address from the outset is the quantity of enzyme that used to load the ITO electrode. Under analytical conditions, with a small electrode, the loading of electroactive FNR can be determined directly from inspection and integration of the signals due to the flavin cofactor observed in cyclic voltammetry;[^27^, ^28^] other enzymes of the cascade may be electro‐inactive, and this is the case for the test system under investigation. In view of the uncertainties regarding the composition of enzymes in the porous layer, which will require detailed investigations exploring many variables, we restricted attention to two simple experiments to determine how catalytic performance depends on the *ratio* of the two enzymes applied to the electrode. Figure 2 shows time courses for the reductive amination of 2‐oxoglutarate to form L‐glutamate, performed with a (FNR+GLDH)@ITO/Ti foil electrode pre‐loaded either side by drop‐casting with FNR and GLDH in different ratios, inserted into a 4 mL stirred electrochemical cell.

All three chronoamperograms show similar timecourses which differed in duration and magnitude. After initiating the reaction by setting the electrode potential (all values quoted adjusted to the SHE scale) to −0.48 V, the rate of conversion remains fairly steady (even increasing slightly) throughout most of the reaction process before dropping rapidly as the reaction nears depletion, coming to rest at zero current. The charges passed for reactions with pre‐mixed FNR:GLDH ratios of 10 : 1, 1 : 1 and 1 : 10 are 6.11, 5.74, 5.77 C, respectively, indicating consumptions of 31.7, 29.7 and 29.9 μmol of reactant, and conversions of 99.1 %, 92.8 % and 93.4 %, respectively. Product conversion was confirmed by NMR spectroscopy (Figure S4).

Cyclic voltammetry was used to observe the qualitative changes occurring as the FNR:GLDH ratio was decreased continuously. An ITO/graphite electrode was pre‐loaded with a high coverage of FNR and then placed in a solution containing 2‐oxoglutarate, NH_4_Cl and TAPS buffer at pH 8.0. After introducing NADP^+^ (50 μM) to the solution, a cyclic voltammogram (CV) was recorded before injecting an aliquot of GLDH stock solution to give a total cell concentration of 8.7 nM. The voltammetry changed from peak‐like to sigmoidal upon introduction of GLDH then increased in current magnitude and changed in shape – the catalytic current eventually showing a linear potential dependence. The results are consistent with the catalytic rate (current) being limited initially by the scarcity of GLDH bound in the pores, then ultimately becoming limited by the rate at which NADPH is electrochemically recycled via the bound FNR. Cyclic voltammograms recorded independently for the three fixed‐ratio conditions used in Figure 2 are shown in Supporting Information (Figure S5): they each reveal a near‐linear dependence of catalytic current on potential, with the current increasing with increase in FNR:GLDH ratio.

**Figure 3:**
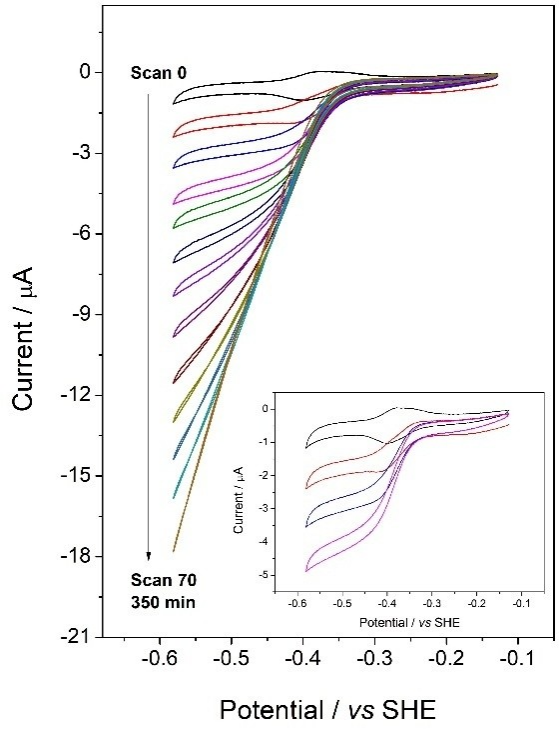
Cyclic voltammograms recorded at different times following the injection of GLDH (to 8.7 nM final concentration) into cell containing 2‐oxoglutarate (20 mM), NH_4_Cl (30 mM) and NADP^+^ (20 μM) in 50 mM TAPS, pH 8.0, 25°C. The stationary ITO/graphite electrode introduced into the solution was preloaded with FNR to give a coverage of 0.2 nmol cm^−2^. The first cycle (scan 0, black trace) was recorded (scan rate 3 mV s^−1^) before injecting the aliquot of GLDH. The inset shows enlarged views of CVs recorded soon after GLDH injection.

Published kinetic data for GLDH measured by conventional means vary greatly with conditions,[^29^, ^30^, ^31^, ^32^] so independent solution assays were made under conditions resembling those in the electrochemical experiments (Supporting information Figure S6). These experiments gave effective values: *k*_cat_=69 s^−1^ per 45 kDa monomer (414 s^−1^ for the functional hexamer) and *K*_m_ (2‐oxoglutarate)=0.50 mM (commensurate with lower values 0.46, 0.64, 0.68 mM reported in the literature[^29^, ^30^, ^31^, ^32^]). Substrate inhibition was evident at comparatively high concentrations of 2‐oxoglutarate with *K*_i_=6.3 mM. Given the initial 2‐oxoglutarate concentration of 8 mM used for the experiment in Figure 2, the solution kinetics data predict that the catalytic rate should increase slightly as 2‐oxoglutarate is consumed, then drop steeply as the 2‐oxoglutarate level finally approaches and passes through the *K*_m_ value. This prediction appears to be borne out well, although the early slow increase should only be observed if the electrode system is sufficiently stable with time. It should also be noted that the tentative interpretation of the voltammetry in Figure 3, i. e. that the cascade is ultimately limited by the rate that FNR recycles NADPH, does not necessarily imply that GLDH is inherently the more active of the two nano‐confined enzymes, since the actual ratio effective in the nanopores remains unknown.

Of the three experiments shown in Figure 2, the one with the 10 : 1 FNR : E2 ratio took the shortest time (6 h) to run to completion, so this ratio was adopted in the remaining experiments. A potential of approximately −0.6 V was used to take advantage of the higher rate, as revealed in the CV experiments.

### 2.4 Relationships in the Rate and Efficiency of NADP^+^ Recycling

**Figure 4:**
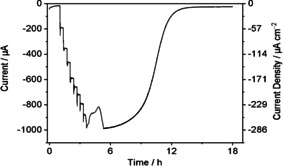
NADP^+^ titration of the reductive amination of 2‐oxoglutarate catalyzed at (FNR+GLDH)@ITO/Ti. Experimental conditions: [2‐oxoglutarate]=40 mM, [NH_4_Cl]=80 mM), NADP^+^ delivered in stepwise additions over range 0–25 μM, buffer: TAPS 0.10 M, pH 8.0; electrode: both sides of 2.5 cm×0.7 cm Ti foil=3.5 cm^2^; temperature: 25 °C, *E* (vs. SHE): −0.59 V; reactor volume: 4 mL; enzymes applied: 17 nmol FNR+1.7 nmol GLDH (10 : 1); loading method: drop‐cast; coulometric yield 94 %.

An NADP^+^ titration was carried out to determine an optimal low cofactor concentration for the pore‐confined coupled reaction. The chronoamperogram shown in Figure 4 (see legend for details) shows the effect of injecting successive quantities of NADP^+^ into the bulk solution (4 mL). Each injection caused an increase in conversion rate, which grew smaller in magnitude with successive additions. The titration was continued until a total concentration of 25 μM NADP^+^ had been introduced.

**Figure 5:**
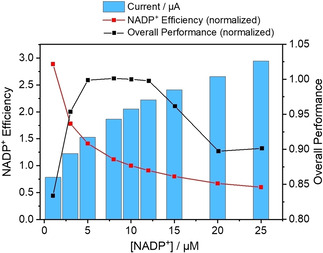
Chart showing the optimization of low concentration NADP^+^. Note: NADP^+^ Efficiency=Current / [NADP^+^]; Overall Performance=NADP^+^ Efficiency×Current. Both quantities are normalized to the value at [NADP^+^]=10 μM.

Figure 5 shows the profile for the increase in current as a function of NADP^+^ concentration (represented as blue bars) along with two other metrics, ‘NADP^+^ efficiency’ and ‘overall performance’. The NADP^+^ efficiency is defined as the current per unit NADP^+^ (normalized to the value at 10 μM); obviously it is highest at lowest NADP^+^ concentration, but this condition would be impractical. Therefore a trade‐off is appropriate: ‘overall performance’ is thus defined as the product of NADP^+^ efficiency and actual current (again normalized to the value at 10 μM). The overall performance was optimized at a NADP^+^ concentration between 5 to 12 μM.

### 2.5 Scale‐Up and Performance

**Figure 6:**
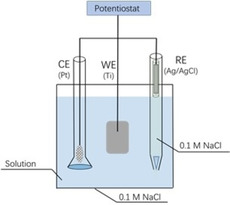
The modular one‐pot reactor used for volumes 80 and 500 mL.

The analytical scale electrochemical cell design adopted a classic three‐electrode system (Figure S7): it holds ca. 4 mL and the active surface area of the Ti foil electrode typically ranges from 3.5 cm^2^ to 14 cm^2^. For reactors holding a much larger volume, a modular system was used, featuring interchangeable side arms for counter electrode (glass frit junction) and reference electrode (Luggin junction) as shown in Figure 6. This design does not require specialized glass parts. The two side arms can be inserted and integrated with any vessel – examples including Schott bottles (50–500 mL) and any large glass jars that can be fitted with an adaptable cap. Multiple ITO/Ti foil electrodes are connected together, and mixing is executed with a large stirrer bar.

**Figure 7:**
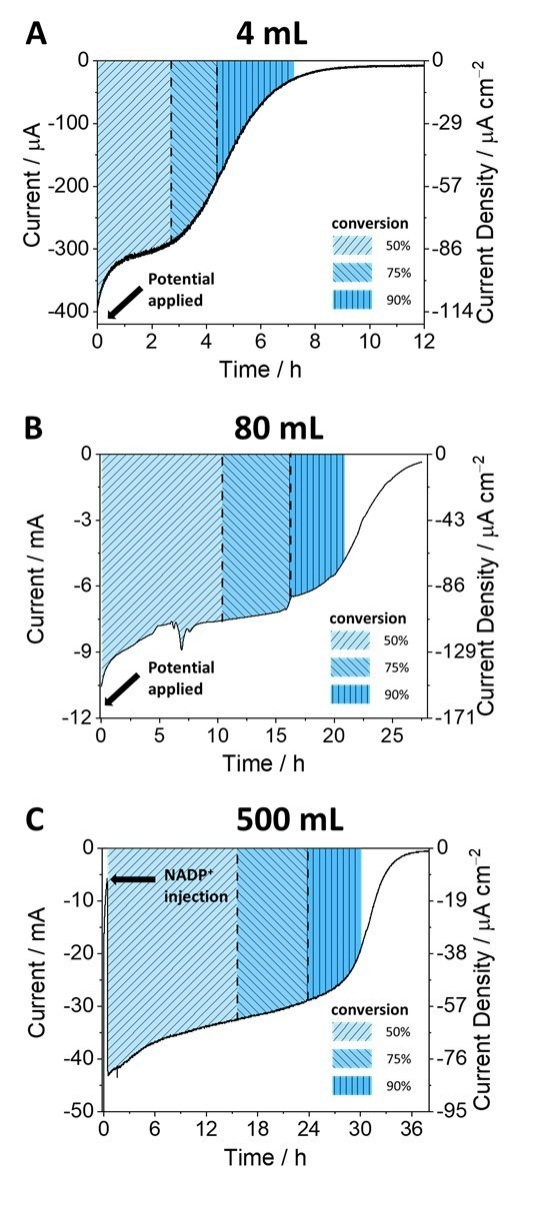
Progress of GLDH‐catalyzed reaction at different scales monitored by chronoamperometry. Conditions: **A) 4 mL reactor**; [2‐oxoglutarate]=8 mM, [NH_4_Cl]=16 mM, [NADP^+^]=20 μM, TAPS 0.10 M, pH=8.0; temperature: 25 °C, *E* (vs. SHE): −0.61 V; **B) 80 mL reactor**; [2‐oxoglutarate]=40 mM, [NH_4_Cl]=80 mM, [NADP^+^]=20 μM, TAPS 0.10 M, pH=8.0; temperature: 25 °C, *E* (vs. SHE): −0.61 V; **C) 500 mL reactor**; [2‐oxoglutarate]=40 mM, [NH_4_Cl]=80 mM, [NADP^+^]=10 μM, No added buffer, initial pH=7.5. Temperature: 25 °C, *E* (vs. SHE): −0.59 V. In all three experiments the enzymes were loaded by the drop‐cast method and agitation was achieved using a stirrer bar at 400 rpm.

Milestone experiments in scaling up are shown in Figure 7, which presents chronoamperograms for the coupled GLDH‐catalyzed reactions undertaken with cell volumes of 4, 80 and 500 mL. The shaded zones represent the progress of the reaction easily calculated from the charge passed to each time point compared to that expected for total conversion.

The reaction carried out in the 500 mL reactor was performed without using any additional buffer, the reactant solution containing only 2‐oxoglutarate and ammonium chloride, adjusted to pH 7.5.

All three chronoamperograms show a similar time course. After initiation, either by injection of NADP^+^ or setting the electrode potential, the current (rate of conversion) remains fairly steady for most of the course of the reaction, and eventually starts to drop sharply as the reactant is depleted. The main difference compared to the small‐scale reactions shown in Figure 2 is that the gradual slight increase in current throughout most of the synthesis is replaced by a slow decrease, possibly reflecting a slightly lower stability when using a more negative potential (−0.6 vs −0.5 V). The total charges passed for the 4, 80 and 500 mL reactors were 6.1, 77, 608 and 3,627 C respectively, from which the coulometric yields were calculated, as presented in Table 1. The chemical yields were determined by NMR spectroscopy (Figure S8), and the product of the 500 mL reaction was purified to give 1.86 g of white crystals (Figures S9–S10). From the respective NMR/coulometry yield ratios 0.996, 1.02, 1.06, it was certain that Faradaic efficiency must be close to 100 %.

**Table 1:** Data of performance for nanoconfined reactors of different sizes.

Cell volume [mL]	Electrode surface area^[a]^ [cm^2^]	NADP^+^ [μM]	Enzyme in loading process [nmol FNR : nmol GLDH]	TTN^[b]^	Reaction time [h]	Current density range [μA cm^−2^]	Yield by coulometry	Yield by NMR
4	3.5	20	7.3 : 0.73	396	8	0–100	30.2 μmol (94 %)	30.1 μmol (93.7 %)
80	73.5	20	348 : 34.8	1,970	27	0–150	3.15 mmol (98.4 %)	3.20 mmol (100 %)
500	525	10	1,092 : 109	3,760	27	0–84	18.8 mmol (94.0 %)	19.8 mmol (99.0 %)

[a] Electrode Dimensions: (1) 4 mL reaction: 1 piece of 2.5 cm×0.7 cm Ti foil; (2) 80 mL reaction: 3 pieces of 3.5 cm×3.5 cm Ti foil; (3) 500 mL reaction: 10 pieces of 7.5 cm×3.5 cm Ti foil; Foils were connected by Ti wire; [b] Total Turnover Number (TTN) of NADP^+^ is defined as the amount of product produced per unit of NADP^+^ used. Electrode potential −0.61 V vs SHE for 4 and 80 mL volumes, −0.59 V for the 500 mL volume.

The entries for current density in Table 1 demonstrate that while the electrode surface area and reactor volume increase (4 mL to 500 mL), the current density remains at a similar level (approximately 90 μA cm^−2^) for >50 % of the reaction in each case, suggesting a successful linear upscale for electrode activity without loss of catalytic efficiency. For the 500 mL reaction, the pH at the end of reaction had shifted only to pH 7.8 which remains within the optimal range for this enzyme. The buffer‐free condition eliminates the interference in product characterization by NMR (see Figure S8C) and facilitates product separation and purification.

## 3 Discussion

The linear scaling of performances of the electrochemical leaf from 4 mL to 0.5 L suggests that this approach should be practical for industrial level synthesis. The low cofactor requirement may stem from the nanoconfinement, as NADPH produced in such a localized environment can be rapidly recycled without leaving the electrode pores, a concentration of 20 μM being commensurate with the *K*_M_ range for both FNR and GLDH.[^33^, ^34^] For comparison, the NAD(P)(H) concentration used in other nicotinamide cofactor regeneration systems usually ranges from hundreds of micromolar to several millimolar.[^35^, ^36^, ^37^, ^38^, ^39^, ^40^, ^41^, ^42^]

The fairly steady current that flows until >75 % of the reaction is complete (as the 2‐oxoglutarate level approaches and passes through its *K*_M_ value) demonstrates that the test system is robust and generally behaves as expected according to comparative enzyme kinetic experiments. The effect of varying the enzyme ratio suggests that the quantity of GLDH required is less important than the quantity of FNR, i. e. GLDH is more efficiently utilized in the confined cascade. This proposal is supported by the CV results (Figure 3) showing that there is a shift to electron‐transfer control as more GLDH binds in the electrode pores. The factors determining the loading of enzymes and their performance are poorly understood at present, and represent an important challenge. Despite this current shortcoming, the ability to monitor the course of the reaction so conveniently would become particularly valuable if the system is extended to drive more complex cascade reactions, all the enzymes of which are immobilized in the nanoconfined state. Obviously, it becomes straightforward to inject more reactant or pause the reaction whenever necessary.

The cost of using the ‘electrochemical leaf’ for synthesis stems mainly from two components, the electrode material and enzymes. For the typical (FNR+GLDH)@ITO/Ti electrode, the electrode material comprises titanium foil and a 1–3 μm layer of ITO nanoparticles (Sigma‐Aldrich <50 nm particle size) available commercially as a powder for the electronics industry. We made a calculation of consumables expenditure appropriate for a university laboratory (details given in Supporting Information). An estimate for the cost of the ITO/Ti support material lies between GBP 0.15/cm^2^ and GBP 0.46/cm^2^ based on current institutional discount and commercial retail prices, respectively. Only the ITO is consumed: the Ti foil can be cleaned by removing the spent ITO and reused an indefinite number of times. The enzyme cost is estimated at between GBP 3/ μmol and GBP 7.40/ μmol based on the FNR preparation process used in this study. The major challenge is to understand how the enzymes are arranged in the electrode pores and establish how loading could be optimized so that only minimal quantities are required. Simple calculations and arguments show that the quantity of enzyme actually undergoing catalytic turnover represents only a small fraction of the enzyme administered in the loading solution, and a large proportion of the enzyme bound in the pores is not contributing to the catalytic current because it is buried too deeply to allow fast exchange with bulk solution.

## 4 Conclusions

This electrochemical cofactor regeneration system using an (FNR+E2)@ITO/Ti‐foil electrode in which all enzymes are nanoconfined has unique properties that underpin its potential for application in enzyme‐catalyzed synthesis, most obviously for complex multi‐step reactions where all the enzymes could be immobilized together in nanoconfined space. The linear scaling between 4, 80 and 500 mL and low cost of materials suggests that reactors well exceeding 5 L might be easily achieved using larger electrodes. Scaling up to an industrial pilot level builds a bridge between delicate laboratory‐scale electrochemistry and application‐oriented chemical engineering and industrial use. In summary, the following aspects are now established:

Advantages

- The ability to run complex cascades in an immobilized state using a simple method of nanoconfinement.
- The ability to energize, control and monitor the reaction continuously in real time, gaining immediate information on rate, yield and effects of adding or removing reagents.
- Scalability – low cost of support and nanomaterials.
- Low cofactor concentrations. NADP^+^/NADPH is only operative in the electrode nanopores.

Challenges

- To gain an understanding of how the enzymes are arranged in the electrode pores and devise highly reproducible, more quantitative loading, leading to improvements in enzyme economy.

## Experimental Section

### Purification of FNR

A vector (aLICator pLATE 51) containing the gene encoding Histagged FNR from *Chlamydomonas reinhardtii* was used to transform *Escherichia coli* cells (BL21 (DE3)) which were subsequently plated on Lysongeny broth (LB) agar containing ampicillin at 100 μgmL^−1^. Positive transformants were selected by resistance to ampicillin. A single colony of this transformation was grown and scaled up, using ampicillin, in a sterile environment. Over‐expression of FNR was induced by the addition of isopropyl β‐D‐1‐thiogalactopyranoside (IPTG). Cells were disrupted using a French press and insoluble material removed by centrifugation. The supernatant was retained and purification of FNR was carried out using a Ni^2+^ HisTrap HP affinity column (GE Healthcare); fractions containing FNR were selected based on the absorbance at 280 nm and 460 nm. The fractions were pooled and concentrated and passed through a desalting column (PD 10 GE Healthcare) to remove imidazole. The enzyme solution was then portioned into single‐use aliquots and flash frozen in liquid nitrogen before storing at ‐80 °C. (see Supporting Information for more details).

### Fabrication of ITO Electrodes

Indium tin oxide‐coated electrodes were prepared by electrophoretic deposition. The EPD suspension was formed by sonicating 0.02 g of ITO nanoparticles (Sigma‐Aldrich <50 nm particle size) in a solution of I_2_ (0.01 g) in acetone (20 mL) for 45 minutes. Two electrodes consisting of Ti foil (thickness 0.127 mm) each connected to a wire by a clip, were placed in the ITO suspension at a separation of 1.5 cm. A voltage of 10 V was applied for 7 minutes across the two electrodes. The Ti cathode at which deposition occurs was then dried thoroughly in air before use. (See Supporting Information for more details).

### Electrochemical Measurements

All electrochemical measurements were conducted using glass cells featuring a three‐electrode system (See Supporting Information for more details). The cell solution was purged with Argon to remove dissolved O_2_ before starting measurements. Enzymes were drop‐cast onto the ITO electrode, incubated for at least 25 minutes, then rinsed with buffer solution and placed in the electrochemical cell. A potentiostat (Multi‐Channel Palmsens) controlled by Multitrace software was used to control the potential and acquire data. Potentials measured using a Ag/AgCl (3 M KCl) reference electrode were converted to the SHE scale by adding 0.211 V.

### NMR Analysis

Samples of the cell solutions were diluted with D_2_O to make a 9 : 1 H_2_O : D_2_O mixture. The ^1^H NMR spectra were recorded on a Bruker AVIIIHD 400 instrument. The peak area corresponding to the product was compared and calculated using (1) a calibration curve obtained from standard solutions of known concentration, and (2) an internal standard with known concentration.

## Conflict of interest

The authors declare no conflict of interest.

## Supporting information

As a service to our authors and readers, this journal provides supporting information supplied by the authors. Such materials are peer reviewed and may be re‐organized for online delivery, but are not copy‐edited or typeset. Technical support issues arising from supporting information (other than missing files) should be addressed to the authors.

Supplementary
