# Supplementary material for: Progress in Scaling up and Streamlining a Nanoconfined, Enzyme‐Catalyzed Electrochemical Nicotinamide Recycling System for Biocatalytic Synthesis
Source: ChemElectroChem. 2020 Nov 20;7(22):4672–8. doi: 10.1002/celc.202001166 (PMC7756331; doi:10.1002/celc.202001166)
Supplement: Supplementary file 1 — Supplementary [file CELC-7-4672-s001.pdf]

ChemElectroChem

Supporting Information

Progress in Scaling up and Streamlining a Nanoconfined, Enzyme-Catalyzed Electrochemical Nicotinamide Recycling System for Biocatalytic Synthesis

Beichen Cheng, Lei Wan, and Fraser A. Armstrong*

Content

Production of Enzymes 3

FNR Expression 3

FNR Purification 3

GLDH Expression 3

GLDH Purification 4

ITO coating 5

Electrophoretic deposition (EPD) method 5

Pasting & Calcination method 6

^{1}H NMR spectrum of GLDH-catalysed reductive amination with different FNR:GLDH ratios 8

Cyclic voltammograms recorded independently for the three fixed-ratio conditions 9

Solution assay for the GLDH-catalysed reductive amination 10

The 4 mL Electrochemical Cell Setup 11

^{1}H NMR spectrum of milestone reactions 12

Isolation of crystalline L-glutamic Acid product 15

Cost of making the (FNR+GLDH)@ITO/Ti electrode in the laboratory 17

i. Cost for ITO coated Titanium Foil 17

ii. Cost for enzyme (FNR) production 17

References 19

Production of Enzymes

FNR Expression

A vector (aLICator pLATE 51) containing the gene encoding N–terminal Histagged FNR from *Chlamydomonas reinhardtii* was used to transform *Escherichia coli* cells (BL21 (DE3))which were subsequently plated on Lysongeny broth (LB) agar containing ampicillin at 100 μgmL^{-1}. Positive transformants were selected by resistance to ampicillin. A single colony was used to inoculate 100 mL LB media containing ampicillin at 100 μg mL^{-1} and grown shaking (200 rpm), overnight at 37 °C. This was used to inoculate 500 mL of LB containing ampicillin at 100 μg mL^{-1} and grown at 37 °C, 200 rpm for approximately 3 hours at which point they were induced by addition of isopropyl β-D-1-thiogalactopyranoside (IPTG) to a final concentration of 1 mM and grown for a further 3 – 4 hours. The cells were then harvested by centrifugation and the pellets resuspended in cold cell resuspension buffer (50 mM HEPES; 150 mM NaCl; 10% V/V Glycerol pH 7.4) and stored at –80 °C until purification.

FNR Purification

The cells were disrupted using a French press at 20 psi and centrifuged at 45000 rpm for 1 hour. The supernatant was retained and purification of FNR was carried out using a Ni^{2+} HisTrap HP affinity column (GE Healthcare); fractions containing FNR were selected based on the absorbance at 280 nm and 460 nm. The fractions were pooled and concentrated using a 4 mL centrifugal filters (Amicon® Ultra–4 Merck) to a final volume of approximately 2 mL. The concentrated protein was passed through a desalting column (PD–10 Ge Healthcare) to remove imidazole, portioned into single use aliquots and flash frozen in liquid nitrogen before storing at –80 °C.

GLDH Expression

Under sterile conditions, competent cells (BL21 (DE3)) were transformed with the HIS–tag *E. coli*

GDH plasmid (previously constructed by first, colony PCR to amplify the GDH gene using E. coli BL21 De3 cells as template, followed by ligation-independent cloning into an N-terminal Histag vector (Thermo Scientific aLICator system #K1251). Colonies were grown on LB agar containing ampicillin at 100 µg mL^{-1}. A single colony was used to inoculate 500 mL LB media containing ampicillin at 100 µg mL^{-1} and also IPTG to a final concentration of 1 mM, and grown 16 h at 37 ℃, 200 rpm.

GLDH Purification

The cells were centrifuged at 6000 rpm (4 ℃) for 30 minutes, resuspended in cell resuspension buffer (pH 7.4) containing 50 mM HEPES, 150 mM NaCl, 10% glycerol and stored at –80 ℃. Upon thawing, the cells were disrupted using a French press at 20 psi and centrifuged at 45000 rpm, (4 ℃) for 1 hour. The supernatant was loaded on a Ni^{2+} HisTrap HP affinity column (GE Healthcare) using Buffer A (50 mM HEPES, 500 mM NaCl, 1 mM DTT, pH 7.4) and Buffer B (50 mM HEPES, 500 mM NaCl, 250 mM Imidazole, 1 mM DTT pH 7.4). A linear (0–100%) imidazole gradient was used; fractions were selected based on the absorbance at 280 nm and also the enzyme activity tested by solution assay using a UV/Vis spectrophotometer (Perkin Elmer, Lambda 19). The fractions were pooled and concentrated using a 10K centrifugal filter (Amicon® Ultra–4 Merck) and dialysed overnight against 1 L of dialysis buffer: 50 mM HEPES, 150 mM NaCl, 1 mM DTT and 10% glycerol. Protein aliquots (20 µL) were flash frozen in liquid nitrogen and stored at –80 ℃.

ITO coating

Electrophoretic deposition (EPD) method

In the EPD method, ITO nanopowder (Sigma-Aldrich < 50 nm) is suspended in a solution of I_2 in acetone and sonicated for at least 45 min. Two electrodes, one of which is the piece of Ti foil to be modified, are held in parallel in the ITO suspension at a close distance apart (ca. 1 cm).[1-2] A 10 V potential is applied between the two electrodes for 5–10 min. The ITO nanoparticles migrate to the cathode and form a thin porous layer. The electrode is dried thoroughly in air before the next stage in which enzymes are bound. The EPD method works particularly well for Ti foil which has a flat face and is easily performed in the lab for surfaces up to 150 cm^2. The average coverage of ITO on Ti foil is approximately 1 mg / cm^2.

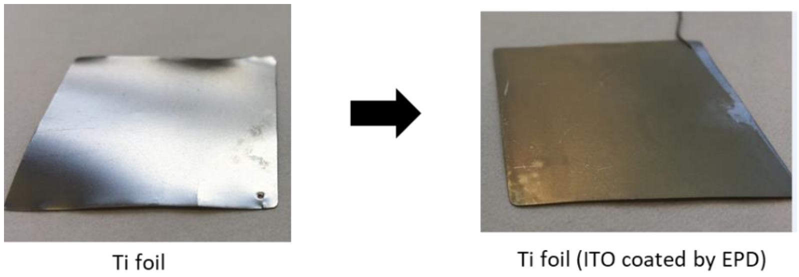

Figure S1. Titanium foil coated by the EPD method

Pasting & Calcination method

The Pasting & Calcination method consists of making an ITO coating paste, painting the paste on the support material, then removing the paste solvent and curing by calcination. The coating paste is made of ITO nanopowders in ethanol with acetic acid, α-terpineol and ethylcellulose. The viscosity and density of the paste solution can be adjusted by changing the ratio of substrates and evaporating the solvent ethanol. The titanium support is painted with a thin layer of ITO paste and then placed in a furnace at 450 ℃ for 30 minutes, after which the ITO layer is strongly attached to the support.

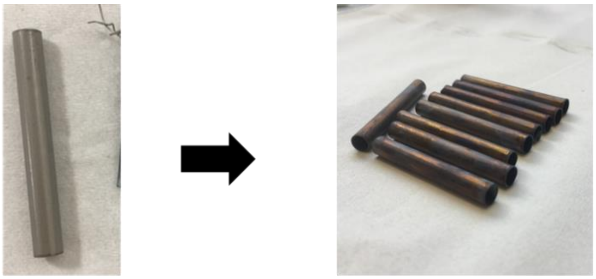

Figure S2 Titanium tube coated by the pasting and calcination method

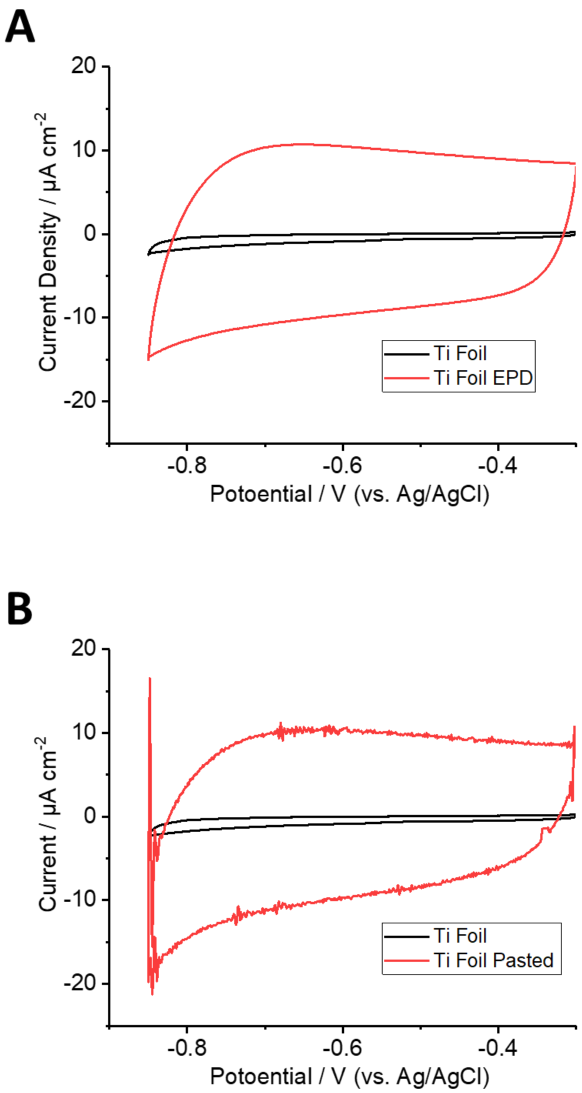

Figure S3. Cyclic voltammograms showing the change in capacitance for electrodes after forming an ITO layer of equivalent depth (typically 3 micron): **A)** ITO deposited by EPD **B)** ITO deposited by pasting and calcination. Experimental conditions: electrode surface area: 14 cm^2 in each case, scan rate: 0.005 V/s, buffer: 100 mM TAPS pH 8.0, temperature: 20 °C.

^{1}H NMR spectrum of GLDH-catalysed reductive amination with different FNR:GLDH ratios

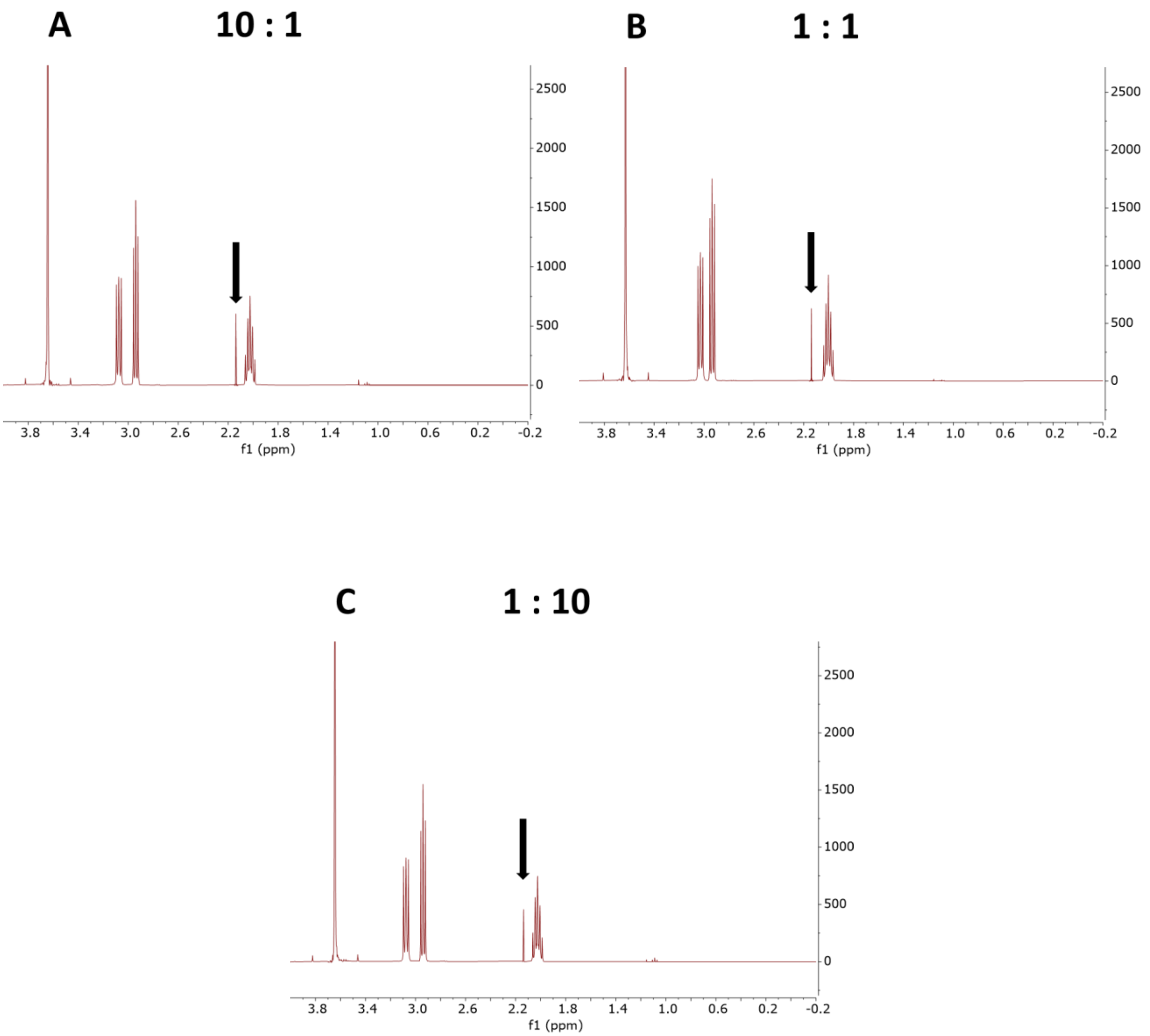

Figure S4 NMR spectra of post-reaction cell solutions for the experiment in Figure 2. Ratio of FNR : GLDH **(A)** 10:1 **(B)**1:1 **(C)** 1:10. The L-glutamate characteristic peak is indicated by the black arrow (δ 2.14 ppm). Product amount was determined by a standard curve method. See Figure S7C for spectrum of 2-oxoglutarate.

Cyclic voltammograms recorded independently for the three fixed-ratio conditions

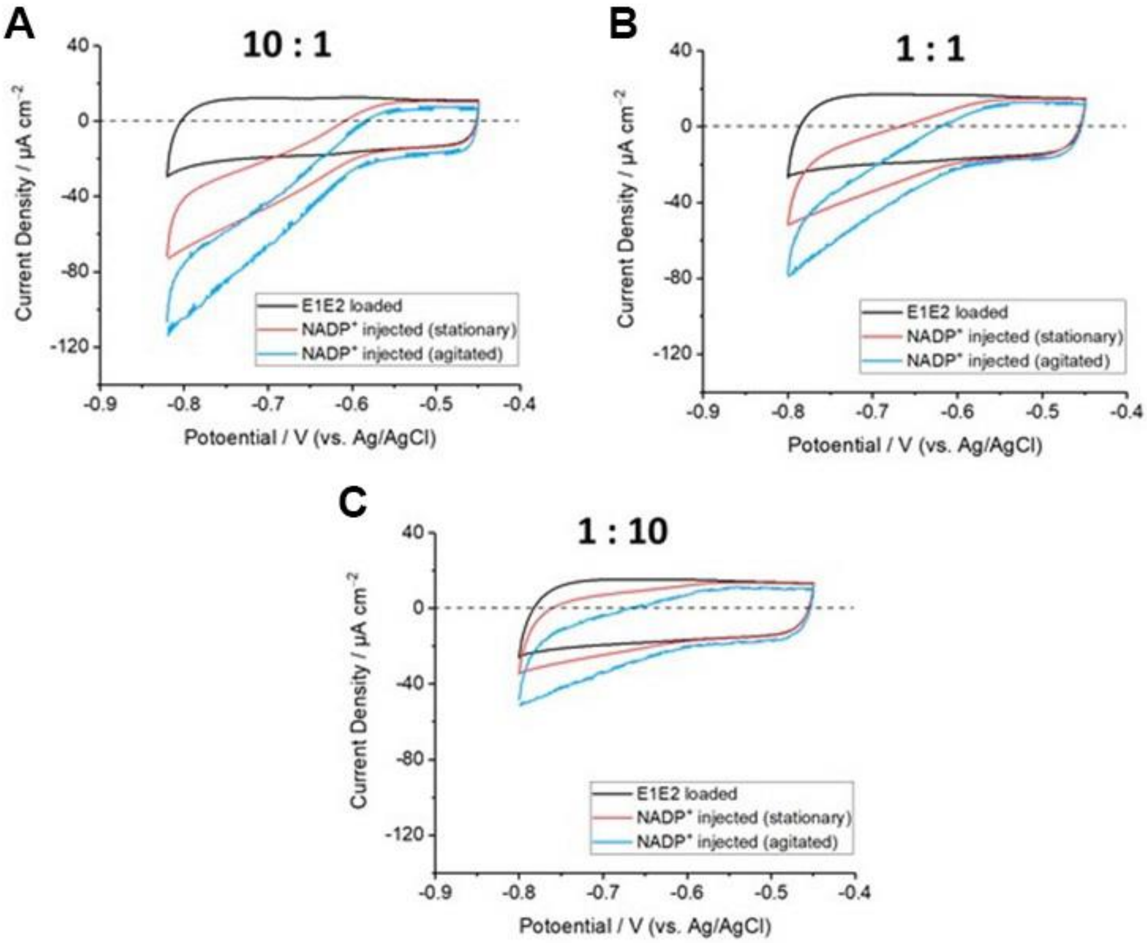

Figure S5 Cyclic voltammograms recorded independently for the three fixed-ratio conditions used in Figure 2. Enzyme applied by drop-casting: 7.3 nmol FNR + 0.73 nmol GLDH (10:1); 4.02 nmol FNR + 4.02 nmol GLDH (1:1); 0.73 nmol FNR + 7.3 nmol GLDH (1:10); Conditions: [2-oxoglutarate] = 8 mM, [NH_4Cl] = 16 mM, [$NADP^+$] = 20 μM; Reactor volume: 4 mL; total electrode surface area (Ti foil): 3.5 cm^2. buffer: 0.10 M TAPS pH 8.0; temperature: 25 ℃, Scan rate: 4 mV s^{-1}; agitation by stirring at 400 rpm.

Solution assay for the GLDH-catalysed reductive amination

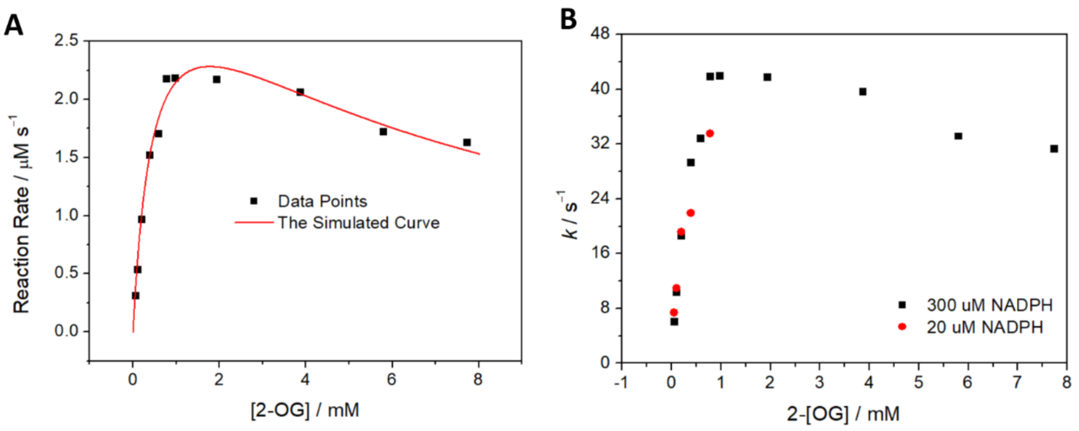

Figure S6 Solution assays of GLDH-catalysed reductive amination with 2-oxoglutarate (2-OG) concentration ranging from 0.049–8 mM under conditions similar to those adopted in the electrochemical experiments. The 'Timedrive' mode of the Perkin-Elmer Lambda 19 UV-vis spectrometer was used. The reaction rate was measured using the tangent of the initial output curve at 340 nm, corresponding to the initial rate of NADPH consumption. (**A)** Measurements carried out when [NADPH] = 300 μM and [GLDH] = 52 nM. The curve was fitted by MATLAB to the equation $v = \frac{v_{\max}[\mathrm{s}]}{\mathrm{K_m} + [\mathrm{s}] + \frac{[\mathrm{s}]^2}{\mathrm{K_i}}}$ in which v is the reaction rate, v_{max} is the theoretical maximum reaction rate achievable, K_m is the Michaelis constant, S is the substrate (herein 2-OG), and K_i is the substrate inhibition constant. The resulting fit gave values for k_{cat} (v_{max}/[GLDH]), K_m, and K_i values of 68.7 s^{-1}, 0.50 mM and 6.3 mM, respectively. **(B)** Comparison between the results in (A) and another set of more limited solution assay results obtained using [NADPH] = 20 μM and [GDLH] = 26 nM. The normalised *k* term in the plot, defined as *v*/[GLDH], allows comparison with the data shown in A. The overlaid plots suggest that at low values of [NADPH] (= 20 μM), the kinetics are similar to those measured at [NADPH] = 300 μM, Experimental conditions: temperature = 22 ± 1℃, $[NH_4]Cl$ = 19.3 mM, total cuvette cell volume = 400 mL, pH = 8.0, 0.10 M TAPS buffer.

The 4 mL Electrochemical Cell Setup

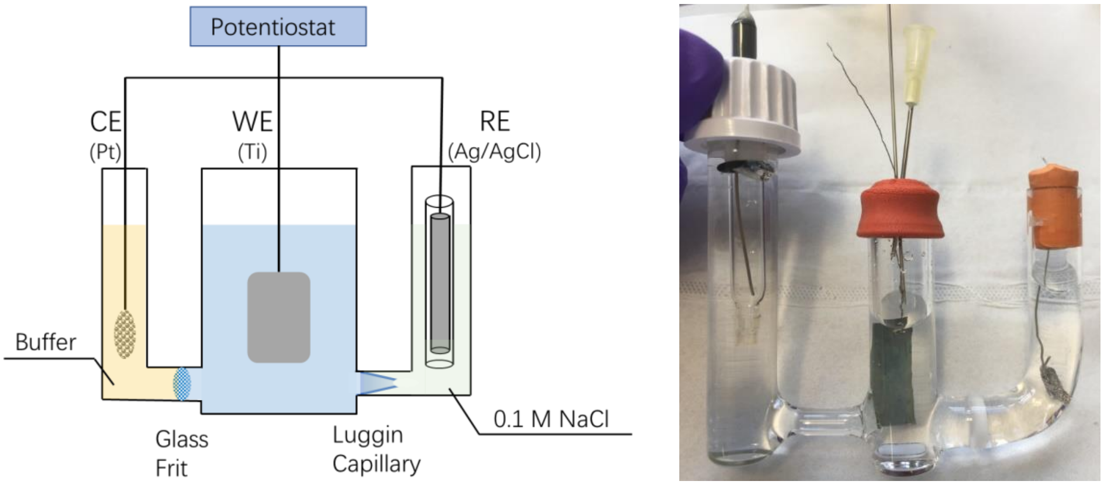

Figure S7 Three-electrode electrochemical reactor.

The conventional three-electrode system consists of three parts. The central section houses the reacting solution and the working electrode. Two side arms are connected to the main part. One side arm with a Luggin capillary junction houses the reference electrode (usually Ag/AgCl in 3M KCl). The other side arm having a glass frit junction houses the Pt mesh counter (auxilliary) electrode. The reference electrode is suspended in 0.1 M NaCl whereas the counter electrode is always in the same buffer as the reaction solution.

^{1}H NMR spectral analysis of milestone reactions

A 4 mL

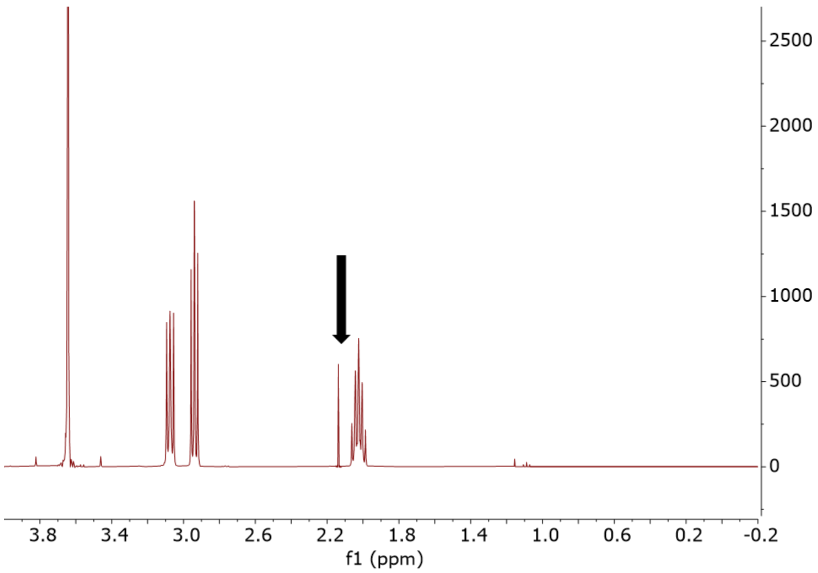

B 80 mL

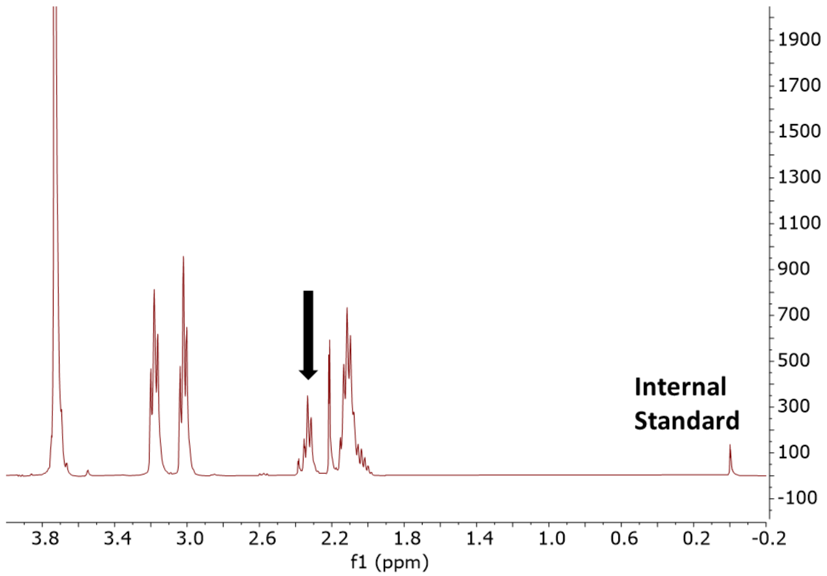

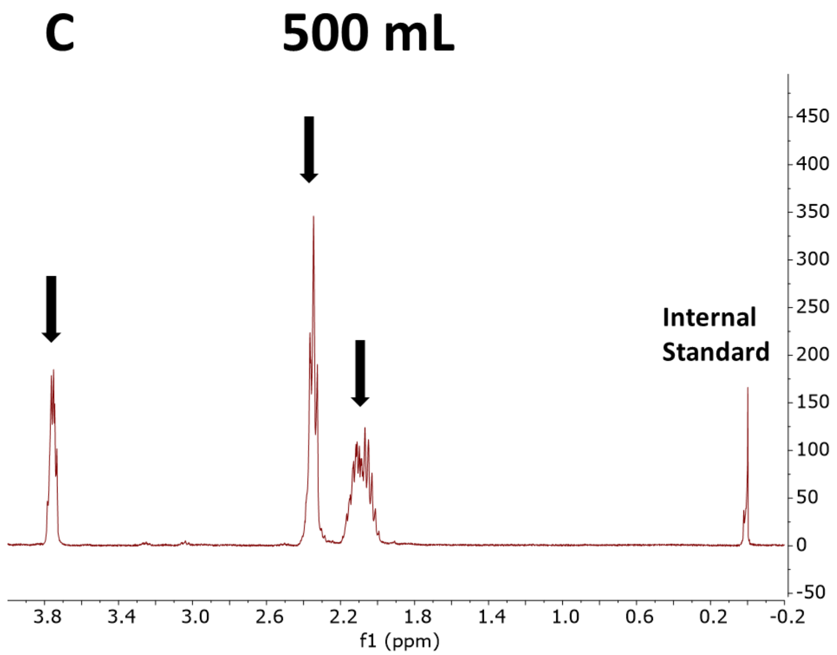

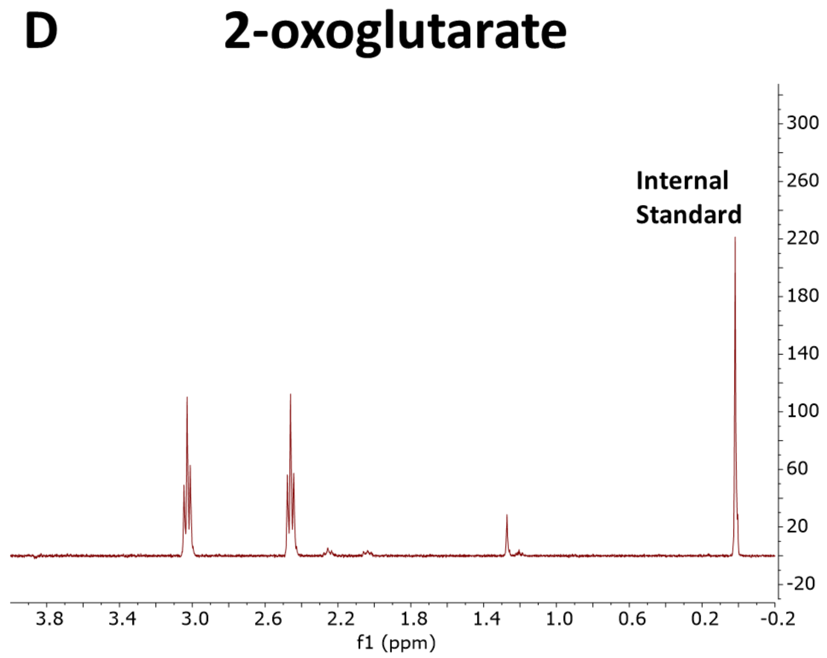

Figure S8 ^{1}H NMR spectra of post-reaction solutions using the reactors in Figure 7. The characteristic L-glutamate peaks are indicated by the black arrows. **(A)** 4 mL (0.10 M TAPS buffer; standard curve method). **(B)** 80 mL (0.10 M TAPS buffer; internal standard method) **(C)** 500 mL (No buffer; internal standard method). **(D)** The reactant 2-oxoglutarate (No buffer; internal standard method) as comparison. Note: Two methods, internal standard method and standard

curve method, were used with different NMR machine settings to calculate the yield of product: this is one of the causes of different spectra appearance. Besides, buffer peaks overlap with several L-glutamate peaks. Therefore the spectrum of the 500 mL reaction with no buffer has more valid characteristic peaks for the L-glutamate product.

Isolation of crystalline L-glutamic Acid product

Pure (>99%) crystal L-glutamic acid is acquired (Figure S8) by crystallization.

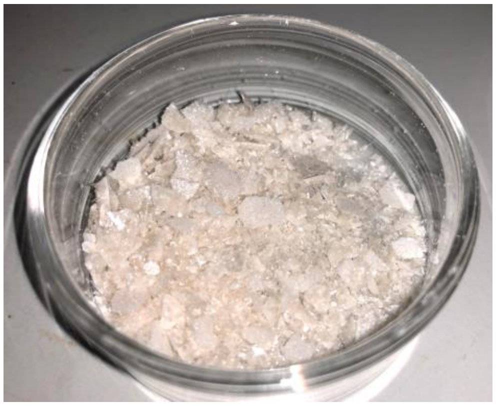

Figure S9 L-glutamic acid crystal after product separation

First, the post-reaction solution is filtered using filter paper. This step removes fine insoluble impurities that consist mainly of dislodged ITO. Decolorization is then performed by adding 10% mass/vol activated charcoal to the solution and stirring for half an hour. The charcoal is then removed by filtration, leaving a clean and colourless solution, the volume of solution reduced from 500 mL to 25 mL by rotary evaporation, and the pH adjusted to 3.2 with HCl. At this pH, L-glutamic acid is in its neutral (acid) form that has very low solubility at low temperature (3.53 g L^{-1} in H_2O at 5.5 °C) and high solubility at high temperature (122.8 g L^{-1} in H_2O at 100 °C). The white suspension is heated to 85 °C to produce a clear, transparent solution. The solution is then cooled down to room temperature and placed in a cold room (4 °C). Finally, the crystalline product is filtered off and dried at 80 °C. The NMR spectrum indicates a purity of over 99% (shown in Figure S9). The mass of pure product (1.86 g) represents a recovery rate of approximately 66% from the product in the cell solution, which could be improved by further work up.

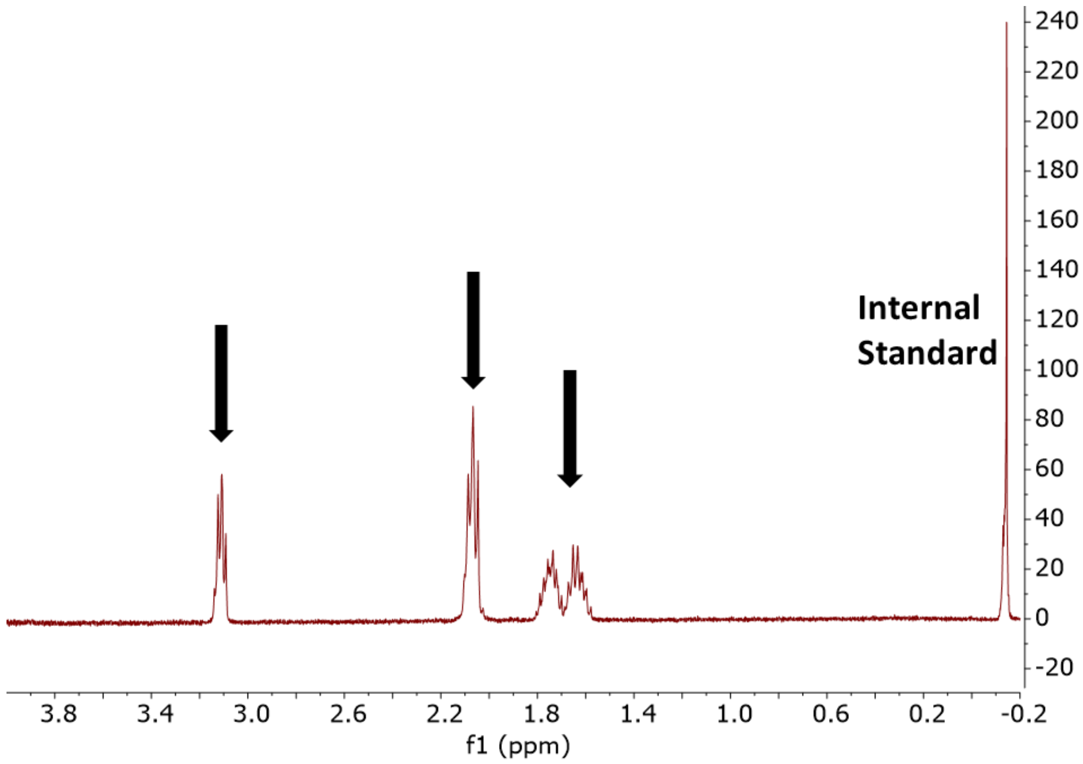

Figure S10 ^{1}H NMR spectrum of L-glutamic acid crystal solution

L-glutamate characteristic peaks are indicated by the black arrow. NMR signal matches the amount (>99%) dissolved in MQ water, indicating a high purity of product crystal.

Cost of making the (FNR+GLDH)@ITO/Ti electrode in the laboratory

For a 10 cm^2 ITO/Ti foil loaded with 20 nmol enzyme,

i. Cost for ITO coated Titanium Foil

electrode surface area: 10 cm^2

Item	Discount price* /GBP	Commercial retail price /GBP	Purchase unit	Use amount	Cost (discount) /GBP	Cost (commercial retail) /GBP
**Ti foil (Reusable)**	29.4	58.8	15cm* 15cm	10 cm2	1.31	2.61
**3.7% HCL (for cleaning)**	8.54	59.42	37% 2.5 L	10 mL	0.0034	0.024
**EtOH (for cleaning)**	3.78	265	2.5L	10 mL	0.015	1.06
**Acetone (for cleaning)**	5.32	105	5L	10 mL	0.011	0.21
**ITO nanopowder**	367.2	612	25g	0.01g	0.147	0.245
**acetone (EPD solution)**	5.32	105	5L	20 mL	0.022	0.42
**Iodine (EPD solution)**	12.67	20.36	2.5g	0.005g	0.025	0.041
**Total cost**					**1.5334**	**4.61**

ii. Cost for enzyme (FNR) production

Typical one batch produces 4 mL of 1.6 mM FNR

Item	Discount price* / GBP	Commercial retail price /GBP	Purchase unit	Use amount	Cost* (discount) /GBP	Cost (commercial retail) /GBP
**Ampicillin**	/	37.48	25g	1g	1.5	1.5
**LB**	/	190.25	5kg	77.5 g	2.95	2.95
**IPTG**	43.12	49	25g	0.714 g	1.23	1.4
**HEPES**	/	100.25	500 g	17.88 g	3.58	3.58
**NaCl**	/	34	1 kg	33.58 g	1.14	1.14
**Centrifuge tube 50mL**	124	226	500	5	1.24	2.26
**Centrifuge tube 15mL**	35.18	137	500	5	0.35	1.37
**Imidazole**	113.05	164	2.5 kg	8.51 g	0.38	0.56
**DTT**	31.68	36	25 g	0.1542 g	0.19	0.22
**EtOH**	3.78	265	2.5L	150 mL	0.225	15.9
**Collecting tubes**	5.94	16.3	1000	50	0.297	0.815
**Ultra-centrifuge tube 10K**	122.98	229	24	1	5.12	9.54
**Bradford dyer**	24.06	61.3	1L	15 mL	0.36	0.92
**BSA protein standard**	52.41	70.1	10 mL	65 μL	0.34	0.46
**UV-Vis cuvettes**	3.99	30.05	100	15	0.6	4.5
**Total cost**					19.502	47.115

* Discount cost is calculated based on the discount prices from department of chemistry, University of Oxford. Research institutes may have discount price for purchasing certain research materials.

The depreciation of research equipment is not taken into calculation and only key consumables are considered. The enzyme preparation protocol can still be further optimized.

Therefore, by the general calculation the enzyme cost is GBP 3.05 / μmol (discount) and GBP 7.37 / μmol (commercial). For a 10 cm^2 ITO/Ti electrode loaded with 20 nmol enzymes (FNR and GLDH in this case), the cost is approximately GBP 1.594 (discount) and 4.76 (commercial).

References

[1] L. Besra, M. Liu *Progress in materials science* 2007, **52**, 1-61.

[2] H. Krüger, A. Knote, U. Schindler, H. Kern, A. Boccaccini *Journal of materials science* 2004, **39**, 839-844.
